# Supplementary material for: A Comprehensive Assessment of Ultraviolet-Radiation-Induced Mutations in Flammulina filiformis Using Whole-Genome Resequencing
Source: J Fungi (Basel). 2024 Mar 20;10(3):228. doi: 10.3390/jof10030228 (PMC10971301; doi:10.3390/jof10030228)
Supplement: Supplementary file 1 [file jof-10-00228-s001.zip › Supplementary Material S8/KEGG annotation/out/64381550635650.os/KO/out_map/map04310.html]

KEGG PATHWAY: Wnt signaling pathway - Reference pathway


|  |  |
| --- | --- |
| **Wnt signaling pathway - Reference pathway** |  |

[
Pathway menu
| Organism menu
| Pathway entry
| Show description
| User data mapping
]

Wnt proteins are secreted morphogens that are required for basic developmental processes, such as cell-fate specification, progenitor-cell proliferation and the control of asymmetric cell division, in many different species and organs. There are at least three different Wnt pathways: the canonical pathway, the planar cell polarity (PCP) pathway and the Wnt/Ca2+ pathway. In the canonical Wnt pathway, the major effect of Wnt ligand binding to its receptor is the stabilization of cytoplasmic beta-catenin through inhibition of the bea-catenin degradation complex. Beta-catenin is then free to enter the nucleus and activate Wnt-regulated genes through its interaction with TCF (T-cell factor) family transcription factors and concomitant recruitment of coactivators. Planar cell polarity (PCP) signaling leads to the activation of the small GTPases RHOA (RAS homologue gene-family member A) and RAC1, which activate the stress kinase JNK (Jun N-terminal kinase) and ROCK (RHO-associated coiled-coil-containing protein kinase 1) and leads to remodelling of the cytoskeleton and changes in cell adhesion and motility. WNT-Ca2+ signalling is mediated through G proteins and phospholipases and leads to transient increases in cytoplasmic free calcium that subsequently activate the kinase PKC (protein kinase C) and CAMKII (calcium calmodulin mediated kinase II) and the phosphatase calcineurin.

|  |  |
| --- | --- |
| Reference pathway | 100% |
